# The *isl2a* transcription factor regulates pituitary development in zebrafish

**DOI:** 10.3389/fendo.2023.920548

**Published:** 2023-02-07

**Authors:** Chen-Yan Yan, Feng-Yao Wu, Feng Sun, Ya Fang, Rui-Jia Zhang, Chang-Run Zhang, Cao-Xu Zhang, Zheng Wang, Rui-Meng Yang, Liu Yang, Mei Dong, Qian-Yue Zhang, Xiao-Ping Ye, Huai-Dong Song, Shuang-Xia Zhao

**Affiliations:** ^1^ Department of Molecular Diagnostics and Endocrinology, The Core Laboratory in Medical Center of Clinical Research, Shanghai Ninth People’s Hospital, Shanghai Jiao Tong University (SJTU) School of Medicine, Shanghai, China; ^2^ Geriatric Medicine Center, Department of Endocrinology, Zhejiang Provincial People’s Hospital, Affiliated People’s Hospital, Hangzhou Medical College, Hangzhou, Zhejiang, China

**Keywords:** *ISL2*, pituitary development, thyroid dysgenesis, transcription factor, zebrafish

## Abstract

**Background:**

ISL LIM homeobox 2, also known as insulin gene enhancer protein ISL-2 (*ISL2*), is a transcription factor gene that participates in a wide range of developmental events. However, the role of *ISL2* in the hypothalamus-pituitary-thyroid axis is largely unknown. In the present study, we characterized the expression patterns of *ISL2* and revealed its regulative role during embryogenesis using zebrafish.

**Methods:**

We used the CRISPR/Cas9 system to successfully establish homozygous *ISL2*-orthologue (*isl2a* and *isl2b*) knockout zebrafish. Moreover, we utilized these knockout zebrafish to analyze the pituitary and thyroid phenotypes *in vivo*. For further molecular characterization, *in situ* hybridization and immunofluorescence were performed.

**Results:**

The *isl2a* mutant zebrafish presented with thyroid hypoplasia, reduced whole-body levels of thyroid hormones, increased early mortality, gender imbalance, and morphological retardation during maturity. Additionally, thyrotropes, a pituitary cell type, was notably decreased during development. Importantly, the transcriptional levels of pituitary-thyroid axis hormones-encoding genes, such as *tshba*, *cga*, and *tg*, were significantly decreased in *isl2a* mutants. Finally, the thyroid dysplasia in *isl2a* mutant larvae may be attributed to a reduction in proliferation rather than changes in apoptosis.

**Conclusions:**

In summary, *isl2a* regulates the transcriptional levels of marker genes in hypothalamus-pituitary-thyroid axis, and *isl2a* knockout causing low thyroid hormone levels in zebrafish. Thus, *isl2a* identified by the present study, is a novel regulator for pituitary cell differentiation in zebrafish, resulting in thyroid gland hypoplasia and phenotypes of hypothyroidism.

## Introduction

The pituitary gland regulates growth, reproduction, and metabolism, and links the nervous and endocrine systems. The pituitary is largely conserved across vertebrates; it is divided into two major parts: the adenohypophysis, which includes the anterior and intermediate lobes, and the neurohypophysis/posterior lobe, which originates from neural ectoderm (1, 2). The adenohypophysis contains multiple glandular cells that are distinguished by the hormones they produce: thyrotropes (thyroid stimulating hormone, TSH), somatotropes (growth hormone, GH), lactotropes (prolactin, PRL), gonadotropes (luteinizing hormone, LH; and follicle-stimulating hormone, FSH), and corticotropes (adrenocorticotrophic hormone, ACTH) (2). The neurohypophysis facilitates the passage of vasopressin and oxytocin into the peripheral blood circulation, which are synthesized in the hypothalamus (2). The intermediate lobe contains melanotropes, which synthesize proopiomelanocortin (POMC), the major precursor of endorphins and melanocyte-stimulating hormone (2).

Multiple transcription factors and genes are involved in pituitary induction, cellular commitment, and cell type specification, including *shha*, *pitx3*, *pit1*, *six1b*, and *eya1* (3–7). *ISL1*, *ISL2*, *LHX3*, and *LHX4* belong to the LIM homeodomain transcription factors that have two tandem cysteine/histidine-rich, zinc-binding LIM domains (8). In particular, several studies have confirmed that *ISL1*, *LHX3*, and *LHX4* play a role in pituitary development (2). Interestingly, *Lhx3* and *Lhx4* participate in the early steps of pituitary ontogenesis in mice and have partially overlapped functions in the development of the anterior pituitary primordium called Rathke’s pouch (9–11). In parallel, *ISL1* is involved in the development and function of thyrotropes as well as gonadotropes (12, 13). Moreover, *ISL1* participates in thyroid and hypothalamus development in addition to pituitary development (14–16).

The hypothalamus-pituitary-thyroid (HPT) axis regulates multiple body functions in all vertebrates *via* endocrine hormones including TSH, thyroxine (T4) and 3,5,3′-triiodothyronine (T3). Despite the fact that thyroid follicles do not form a compact gland but remain loosely dispersed along the pharyngeal midline, T4 has negative feedback effects on the release of TSH in the pituitary of zebrafish, an ideal model system in endocrine research due to its high conservation of molecular mechanisms involved in organogenesis, hormone transport and metabolism, as well as hormone action in target tissues (17, 18). Nevertheless, unlike in mammals, the role of the thyrotropin releasing hormone in the regulation of TSH release in fish is less well established (19). Due to the absence of a portal system between the hypothalamus and the pituitary, the zebrafish hypothalamus directly innervates the pituitary (20). Notably, *ISL2* and *ISL1* are both members of the Islet-1 family, and share 72% protein sequence identity in mice (21). These two genes may have similar functions given their close structural similarities. However, the role of *ISL2* in pituitary development has not been clarified yet. Thus, we investigate the role of *ISL2* in the development and function of the HPT axis.

In the current study, we utilized the zebrafish model to characterize the role of *ISL2* in the development of HPT axis. We found that *ISL2*, as a transcription factor, is a critical factor for the correct differentiation of pituitary sub-types and that it affects thyroid development through reducing proliferation rather than affecting apoptosis.

## Materials and methods

### Zebrafish husbandry

The Tübingen strain of zebrafish (Danio rerio) used in this study was maintained at 28.5°C with a light-dark cycle of 14 hours/10 hours and at a stocking density of 6-8 fishes/L. The zebrafish were fed twice a day with Paramecium, Brine Shrimp, and flake food. Food concentration depends on zebrafish body weight and the purpose for which the animals are kept. The embryo stages are expressed in hours post-fertilization (hpf) at a standard temperature. Fertilized eggs were collected *via* natural spawning and raised in egg water (0.06 g/L Instant Ocean Sea Salt and 22.2 ug/L methyl blue). The Tg (*tg*: egfp) transgenic line that specifically expresses green fluorescent protein (EGFP) in thyroid cells was used for this study. The Shanghai Jiao Tong University School of Medicine’s Institutional Animal Care and Use Committee examined and approved all animal protocols.

### Generation of the *isl2a* and *isl2b* CRISPR knockout zebrafish lines

Knockout fish lines were generated *via* the CRISPR/Cas9 technique (22, 23). CRISPOR (http://crispor.tefor.net/) was used to design an *isl2a* sgRNA targeting exon 3 near the LIM2 domain (5’-GGCGGACCACGGACTGCTAA-3’). Similarly, the *isl2b* sgRNA, targeting exon 2 in the LIM2 domain (5’-GGGAGACGCGCAGGATGTAC-3’), was synthesized in the same way as *isl2a*. Then, using the MEGA script™ T7 Transcription Kit (Ambion), sgRNAs were transcribed and purified using the mirVana™ miRNA Isolation Kit (Ambion). Cas9 mRNA was transcribed with the SP6 mMESSAGE mMACHINE™ Kit (Ambion) and purified with the AxyPrep DNA Gel Extraction Kit (Axygen). A total of 50 pg of sgRNA and 300 pg of Cas9 mRNA (2 nL volume) were injected into single-cell stage fertilized wild-type embryos.

Sanger sequencing was used to confirm the mutation at the target location. The surviving sgRNA/Cas9-injected embryos (F0 founders) were nurtured to adulthood and outcrossed with wild-type adults to produce the F1 generation. The knockout line was established using an F0 founder with germline transmission and a high rate of indels. F1 generation embryos were brought to adulthood, fin clipped, and sequenced. Individuals carrying the same variant were identified and pooled together. All experiments were carried out on embryos derived from F2 or F3 offspring.

A whole larva or fin clip was placed in a separate tube with 50 μL of lysis buffer (1M Tris-HCl, 0.5μM EDTA, 10% Tween and 10% NP40) to extract genomic DNA in order to determine the genotype of the larvae. Lysis was initiated at 95 °C for 10 min, then the samples were incubated at 55°C overnight before being heated to 98°C for 10 min. Lysed samples were genotyped by PCR amplification of a region of interest (containing the *isl2a* 13bp insertion or *isl2b* 5bp deletion) using Taq DNA Polymerase (Lifefeng) and specific primers (**Table S1**). The samples were subjected to Sanger sequencing using the same primers used for amplification. All larvae were genotyped to generate lines and for all phenotypic characterizations.

### Whole-mount *in situ* hybridization

WISH was undertaken as previously described (24) using digoxigenin-labeled probes (Roche). The probes were made as previously described (25, 26). The target gene’s coding sequence fragment was amplified and cloned into the pGEM^®^-T vector (Promega) using wild-type strain cDNA. For anti-sense DIG-labeled RNA probes, cloned DNA was linearized with various endonuclease enzymes before being produced with SP6 RNA polymerase or T7 RNA polymerase using the DIG RNA labeling kit (all Roche). In zebrafish, *shha* (3), *pitx3* (4), *lhx4* and *lhx3* (27) are involved in the determination of the fate of pituitary precursor cells. *Pit1* and *six1b* are required for lineage-specific differentiation of the pituitary; the *pit1* lineage depends on *pit1* (5), while the non-*pit1* lineage depends on the *eya1*/*six1b* protein complex (6). Pituitary precursors differentiate into specific cell types with markers, including *gh* (for somatotropes), *tshba* (for thyrotropes), *cga* (for thyrotropes and gonadotropes), *prl* (for lactotropes) and *pomca* (for corticotropes and melanotropes) (28). Thus, *isl2a*, *isl2b*, pituitary cell markers (including *shha, pitx3, lhx4, lhx3, pit1, six1b, gh, prl, tshba, cga*, and *pomca*), and thyroid cell markers (including *tg*) were synthesized. All primer sequences are described in **Table S2**. All DNA constructs were verified by sequencing.

Embryos were fixed in 4% paraformaldehyde, rinsed in 1× phosphate-buffered saline with 0.1% Tween^®^ 20 detergent (PBST), destained in 5% hydrogen peroxide, washed in 25-100% methanol successively and stored in 100% methanol at -20 °C until needed. Embryos were rinsed with 75-25% methanol followed by 1× PBST on the first day of the WISH procedure. Embryos were fixed again with 4% paraformaldehyde and washed with 1× PBST after being treated with proteinase K (Sigma) according to the developing stages for permeabilization. Embryos were hybridized with the RNA probes in the hybridization mix solution overnight at 68°C. On the second day, embryos were blocked with 10% fetal bovine serum (Thermo) and incubated with anti-digoxigenin-alkaline phosphatase Fab fragments (Roche) overnight at 4°C after being washed with 2× saline sodium citrate, 0.1% Tween 20 (SSCT)/50% deionized formamide, 2× SSCT and 0.2× SSCT at 68°C. Embryos were produced on the third day using 5-bromo-4-chloro-3’-indolyphosphate p-toluidine salt (BCIP)/nitro-blue tetrazolium chloride (NBP) substrate (Roche). Staining was developed and stopped before the background signals started to appear.

### mRNA extraction and qRT-PCR analysis

Total RNA from 3 days post-fertilization (dpf) and 5dpf *isl2a^+/+^*, *isl2a^+/-^* and *isl2a^-/-^* larvae, was extracted utilizing TRIzol (Ambion, Life Technologies). Reverse transcription of total RNA (1ug) to single-stranded cDNA was carried out using the PrimeScript™ RT reagent Kit with gDNA Eraser (Perfect Real Time, Takara) and further diluted 1:10. Next, we use the Quant Studio 12K Flex Real-Time PCR System (ABI, USA) to perform qRT-PCR. The primer sequences for real-time detection of target gene mRNA are shown in **Table S3**. The reaction mixture, containing diluted cDNA template, primers, and 2× TB Green Premix (Takara), was amplified under cycling conditions based on the manufacturer’s protocol. Finally, we analyzed the generated data using the corresponding software. The transcripts of all genes were normalized against the housekeeping genes, such as elongation factor 1-alpha (*ef1α*) and beta-actin (*actb*). To determine the relative levels of mRNA expression between experimental samples and controls, we used the ΔΔCq method. At the same time, the data comprising the results were from at least two separate experiments run in triplicate.

### Morphological studies in knockout zebrafish

Daily counts of *isl2a* ^+/+^, *isl2a* ^+/-^ and *isl2a* ^-/-^ larvae were made, and notable dysmorphologies such as edema, head, and eye malformations were checked for. For head, ear, eye and female vent size measurements, *isl2a* ^+/+^, *isl2a* ^+/-^, and *isl2a* ^-/-^ larvae were anesthetized in 160 mg/L tricaine methane sulfonate (MS222, Sigma) and positioned in 3% methylcellulose. Lateral images of the head and the whole body of 3dpf and 5dpf larvae were acquired using a Research Stereo Microscope SMZ25 equipped with a Microscope Camera DS-Ri2 (Nikon) and NIS-Elements BR 4.50.00 software. Measurements were manually performed in a blinded manner using ImageJ software. Body length from the anterior tip of the snout to the base of the posterior caudal fin was measured. Heart rate was measured using 30 second video recordings. The body lengths of 14 dpf, 41 dpf, 77 dpf and 7.5 months post-fertilization (mpf) zebrafish were measured by a vernier caliper.

### Histological analysis of gonadal tissues

To prepare tissue sections, 42 dpf fishes were anesthetized with 160 mg/L tricaine methane sulfonate (MS222, Sigma), and their gonads were carefully dissected. In this study, gonads from three fish per group were used for the histological analysis. Samples fixed in paraformaldehyde were dehydrated in ethanol, cleared in xylene, embedded in paraffin, cut into 5μm sections, stained with hematoxylin-eosin and observed under a microscope. The staging of ovary and testes development was based on the cellular structure (29, 30).

### TUNEL and EdU assays

For immunofluorescence staining, the embryos were fixed with 4% paraformaldehyde, permeabilized with 0.5% Triton X-100, and blocked with 5% bovine serum albumin. The embryos were then incubated with primary antibodies against EGFP (1:200; Abcam) at 4°C overnight, followed by incubation with an anti-rabbit IgG secondary antibody/Alexa-Fluor 488 (1:500; Life Technologies). After completion of the immunofluorescence staining, proliferation and apoptosis were analyzed by EdU and TUNEL assays using a Click-iT EdU Imaging Kit (Invitrogen) and a One Step TUNEL Apoptosis Assay Kit (Beyotime) according to the manufacturer’s protocol, respectively. Finally, the fluorescent signals were detected by confocal microscopy. The number of EdU- or TUNEL- positive thyroid cells was calculated by delineating the region of *tg* expression using ImageJ software. Stacks were recorded using a 20× objective plus 2× zoom (Nikon C2+ confocal system; Nikon), and the images were processed utilizing Adobe Photoshop CS2.

### Measurement of hormone levels

Whole-body thyroid hormone levels (T4 and T3), gonadotropic hormone concentrations (FSH and LH) in gonads were measured using commercial ELISA kits (Cloud-Clone Corp., Wuhan, China, and ELK Biotechnology Corp., Wuhan, China, respectively) following the manufacturer’s instructions (31, 32). The numbers of individuals used in thyroid hormone levels measurement for the time point of 44dpf was one fish as a sample. The whole gonadal tissues of two fish as a sample to extract gonadotropic hormone at 42dpf. Briefly, each sample was completely homogenized by continuous vortex for 1-2 min at 65 Hz on ice, and centrifuged at 5000 × g for 10 min at 4°C. Next, supernatants were collected and stored at -80 °C for the measurement. As for the levels of testosterone and estradiol in the gonads, a high-performance liquid chromatography System (LC-30A, SHIMADZU, JAPAN) coupled with a triple quadrupole mass spectrometer (QTRAP6500, SCIEX, USA) method (HPLC-MS) was used for multiple reaction monitoring (MRM) analysis (33). The detection of testosterone (289.2/97.3) was operated in positive mode and estradiol (271.2/145) in negative mode. The instrument parameters were as follows: capillary voltage: 5500 V (+)/-4500 (-). MRM declustering potential, entrance potential, collision energy, collision cell exit potential were optimized for each metabolite by flow-injection analysis mode. The sample preparation was performed as follows: the whole gonadal tissues of around 4 mpf zebrafish were completely homogenized with 0.4 ml 0.1× phosphate-buffered saline solution, then centrifugation at 15,000 rpm for 20min at 4°C. The supernatant was removed, added to a threefold precooled methanol solution, and stored at -20°C for 30 minutes. Then centrifugation again at 15,000 rpm for 10min at 4°C, the supernatant was removed and blow-dried under nitrogen, and resuspended in 100ul isopropanol/acetonitrile/water solution (30:65:5). After centrifugation, the supernatant was immediately used for hormone analysis.

### Statistical analysis

The data of testosterone and estradiol is presented as the mean ± SEM, other data is presented as the mean ± SD or percentages. For data failing the normality test, the pairwise statistical significance was established using Student’s unpaired t-test or Mann-Whitney test, and multiple comparisons were determined using one-way ANOVA with Tukey’s test. All statistical analyses were performed using GraphPad Prism6 software (GraphPad, San Diego, CA, USA). The significant differences were shown at * P < 0.05, ** P < 0.01, *** P < 0.001 and **** P < 0.0001.

## Results

### *Z*ebrafish *ISL2* orthologue gene knockout using CRISPR/Cas9

**Figure 1:**
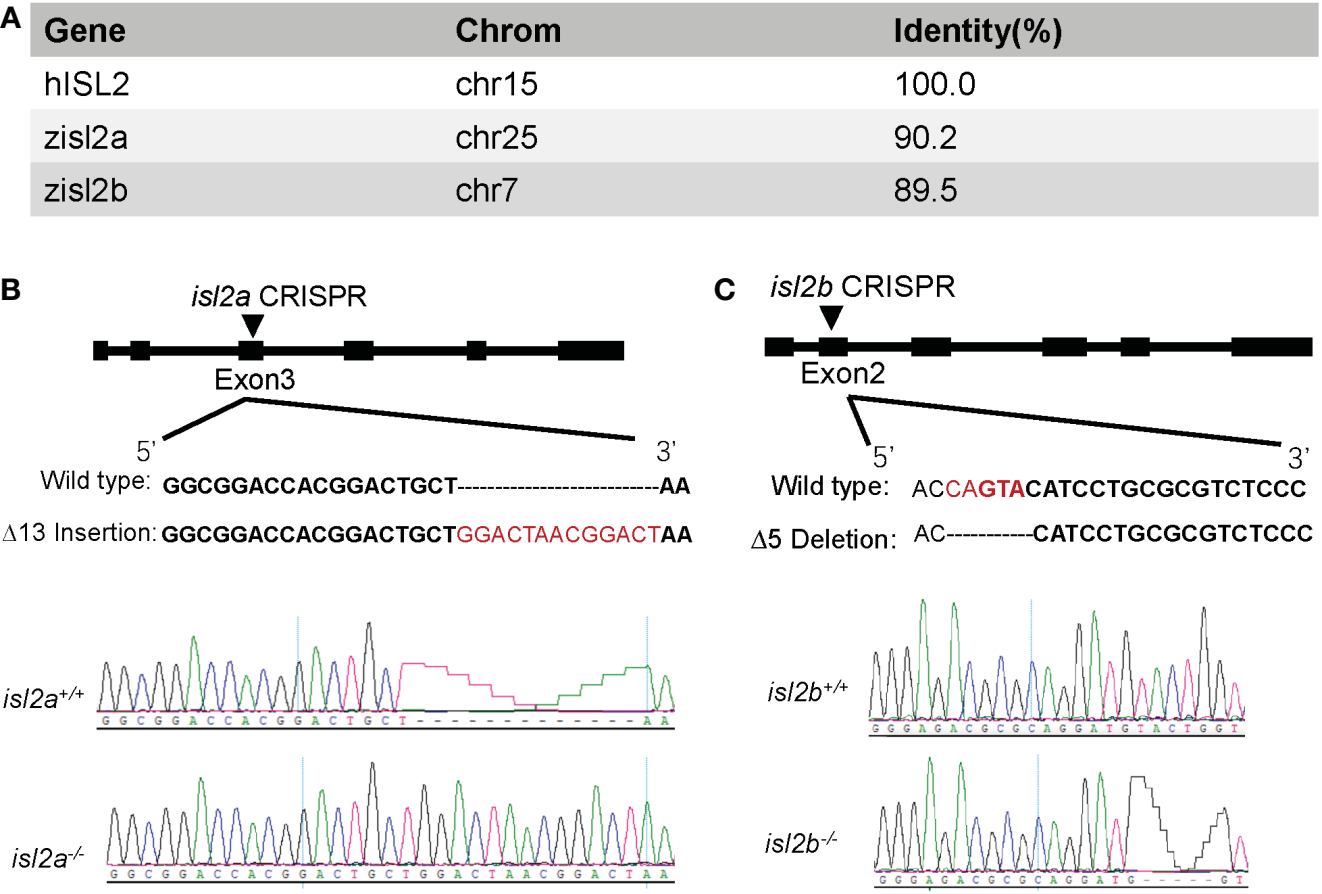
Generation of *isl2a* and *isl2b* knockout zebrafish utilizing CRISPR-Cas9 technology. **(A)** Amino acid identity between zebrafish and human *ISL2* proteins was performed using UCSC Blat software. **(B)** The *isl2a*-CRISPR-target site (black bold words) was designed in exon3 of *isl2a* and the CRISPR/Cas9-induced insertion of 13 nucleotides (red words) was selected for further investigation. **(C)** In the case of *isl2b*, the CRISPR-target site (bold words) was designed in exon2, and the induced deletion of five nucleotides (red words) was selected for further investigation. Genotypes, including wild-type and homozygotes, were analyzed and shown in the bottom panel. Both genotypes were predicted to produce truncated proteins.

The zebrafish genome encodes two single *ISL2* orthologues (*isl2a* has 90.2% identity and *isl2b* has 89.5% identity at the amino acid level compared to the human *ISL2* protein) with a highly conserved domain (**Figure 1A**). To examine potential functional effects of *ISL2* loss-of-function *in vivo*, we generated *isl2a* and *isl2b* knockout models using CRISPR/Cas9 technology in zebrafish. The *isl2a*-CRISPR-target site was designed in exon 3 of *isl2a*, and CRISPR/Cas9-induced insertions of 13 nucleotides were chosen for further *isl2a* mutant line generation and maintenance (**Figure 1B**). The target site for *isl2b* was designed in exon 2 of *the gene*, and CRISPR/Cas9-induced deletions of five nucleotides were selected for further generation and maintenance in the *isl2b* mutant line (**Figure 1C**). At 3 dpf, qRT-PCR analysis validated an almost complete lack of *isl2a* mRNA in *isl2a* ^-/-^ larvae and approximately half of the transcript was present in *isl2a* ^+/-^ larvae, which demonstrates the efficacy of the *isl2a* knockout. Both mutants were verified *via* Sanger sequencing and were predicted to produce truncated proteins.

### *Isl2a* loss-of-function variant reduces subtypes of pituitary cells

**Figure 2:**
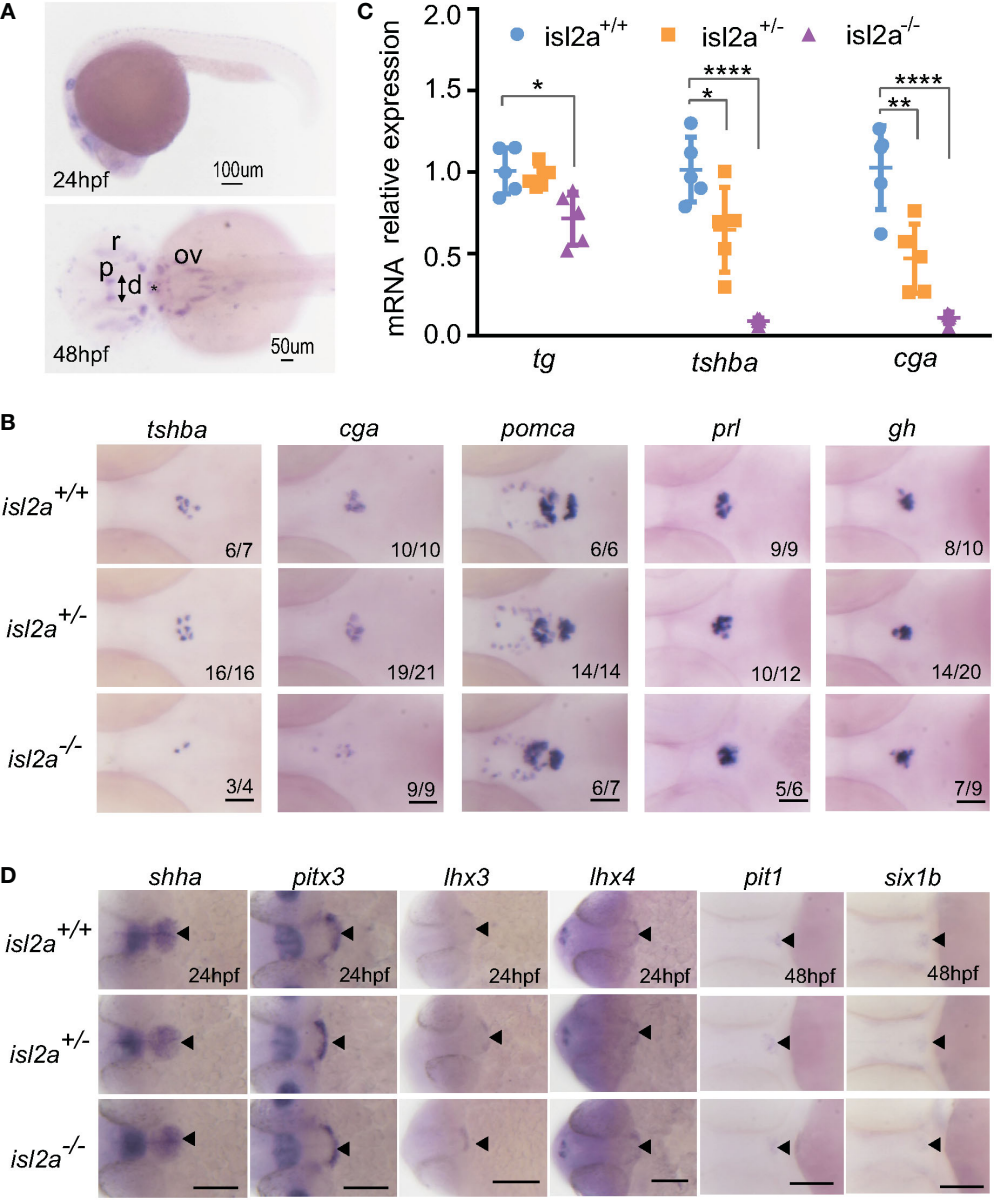
Effect of *isl2a* knockout on the expression of genes involved in pituitary development *via* whole-mount *in situ* hybridization. **(A)** Spatiotemporal expression patterns of *isl2a* by whole-mount RNA *in situ* hybridization at 24 and 48 hpf. Scale bars = 100 µm/50 µm. Asterisks (*) indicate the position of midbrain-hindbrain boundary. p, pineal gland; r, retinal; ov, otic vesicle; d, diencephalon. **(B)** Knockout of *isl2a* resulted in reduced *tshba* and *cga* expression, while the expression of *pomca*, *prl*, and *gh* was not changed in larvae at 3 dpf. Pituitary precursors differentiate into specific cell types with markers, including *gh* (for somatotropes), *tshba* (for thyrotropes), *cga* (for thyrotropes and gonadotropes), *prl* (for lactotropes), and *pomca* (for corticotropes and melanotropes). All images are dorsal views with the head pointing towards the left. Scale bar = 50 µm. Numbers indicate the ratio of embryos with the shown phenotype. **(C)** qRT-PCR analysis demonstrates the expression of total *tg*, *tshba* and *cga* in *isl2a^+/+^*, *isl2a^+/−^* and *isl2a^−/−^* larvae at 3 dpf. Data is represented as mean ± SD (n = 5, 15 fish per tube). *P<0.05, **P<0.01, ****P<0.001. **(D)** Ventral views of embryos at 24 hpf show no differences in *shha*, *pitx3*, *lhx3* and *lhx4* expression in *isl2a* mutants. Dorsal views of embryos at 48 hpf show no difference in gene expression, indicating specification of pit-1 lineage (*pit1*, for somatotropes, thyrotropes, and lactotropes) or non-pit-1 lineage (*six1b* for corticotropes, gonadotropes, and melanotropes). Thus, pituitary induction and lineage specification were unaffected in *isl2a* mutants. Scale bar = 100 µm. The black triangle indicates gene expression in the adenohypophyseal placode.

As we all know, *isl2a* is expressed in a diffuse anterior area, as well as in the pineal gland and ventral hindbrain at 24 hpf (**Figure 2A**). However, the expression of *isl2a* was limited to the pineal gland and subsets of the retinal and otic cells at 48 hpf, with modest expression in the diencephalon at 48 hpf (**Figure 2A**). To investigate the potential effects of *ISL2* on HPT axis development, we performed WISH to analyze the mRNA expression levels of several cell marker genes, including *tshba* (encoding the beta subunit of TSH) and *tg* (encoding thyroglobulin). Interestingly, the thyrotropes were reduced in *isl2a* ^-/-^ embryos compared to *isl2a* ^+/+^ and *isl2a* ^+/-^ embryos with reduced expression of *tshba* and *cga* (encoding alpha subunit of TSH, LH, and FSH) at 3 dpf (**Figure 2B**). Somatotropes and lactotropes, on the other hand, showed no alternations in *isl2a* ^-/-^ embryos, as indicated by the expression of their corresponding markers, *gh* and *prl*, respectively (**Figure 2B**). In addition, no significant difference in corticotropes and melanotropes among the *isl2a* ^+/+^, *isl2a* ^+/-^ and *isl2a* ^-/-^ embryos was observed, as indicated by *pomca* expression (**Figure 2B**). Similarly, *tshba*, *cga*, and *tg* transcript levels in *isl2a* ^-/-^ embryos were reduced to 8.62%, 10.50%, and 71.00% of the levels in *isl2a* ^+/+^ embryos, respectively, according to qRT-PCR analyses (**Figure 2C**).

For *isl2b*, it is expressed in zebrafish several structures, including midbrain, hindbrain and pharyngeal arch at 24 hpf, and retinal, one endoderm primordium, and branchial arches at 48 hpf (**Figure S1A**). No significant differences in *tshba* or *tg* transcription levels were found among the *isl2b* ^+/+^, *isl2b* ^+/-^ and *isl2b* ^-/-^ embryos (**Figure S1B**). Interestingly, we found genetic compensation in the *isl2b* mutants. Compared to *isl2b* ^+/+^ embryos, the expression of *isl2a* in *isl2b* ^+/-^ and isl2b^-/-^ embryos were significantly increased by 26.81% and 51.56%, respectively (**Figure S1C**). Genetic compensation may contribute to the lack of differential expression of the *tshba* and *tg* transcripts. Furthermore, in the offspring of *isl2a^+/-^isl2b^+/-^* mating, we found reduced *tshba* and *cga* transcripts in *isl2a^-/-^* zebrafish, but no phenotype in *isl2b^-/-^* zebrafish (**Figure S1D**).

We next investigated early pituitary development. As shown in **Figure 2D**, the overall size of the pituitary, as analyzed by *shha*, *pitx3*, *lhx3*, and *lhx4* expression, was not affected in *isl2a* mutants at 24 hpf. These results suggest that overall cell numbers in *isl2a* mutants were normal, indicating that *isl2a* is not necessary for early development of the pituitary. To investigate early cell fate specification in the pituitary of *isl2a* mutants, the expression levels of *pit1* and *six1b* were analyzed. There were no noticeable variations in *pit1* or *six1b* expression in *isl2a* mutants at 48 hpf compared to controls (**Figure 2D**). Thus, these findings suggest that *isl2a* wasn’t essential for the early formation of the pituitary or for the specification of pit1 and non-pit1 lineages.

### Effects of low TSH level on thyroid morphology and function

**Figure 3:**
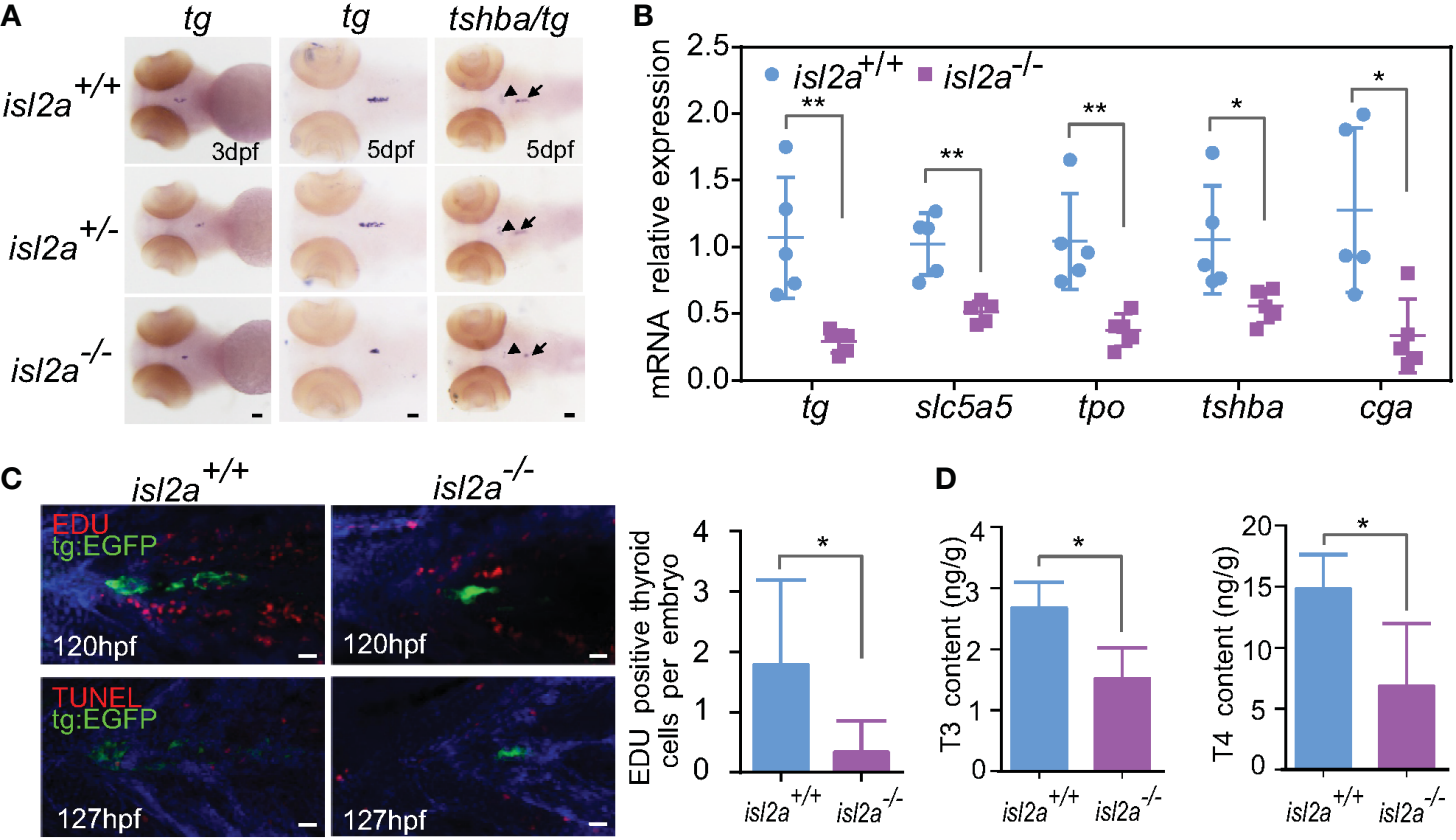
*Isl2a* mutants show reduced thyroid function. **(A)** The assessment of *tg* expression in *isl2a* mutants at 3 dpf and 5 dpf *via* whole-mount *in situ* hybridization. At 5 dpf, *isl2a^-/-^* zebrafish had lower expression of *tg* and *tshba* as denoted by black arrows and arrowhead, respectively. Scale bar = 50 µm. **(B)** qRT-PCR analysis of the total expression of *tg*, *slc5a5*, *tpo*, *tshba*, and *cga* in *isl2a^+/+^* and *isl2a^-/-^* larvae at 5 dpf. Error bars represent ± SD (n = 5, 15 fish per tube). **(C)** Both EGFP (green) and EdU immunoreactivity (red) were present in the thyroid of 120 hpf Tg (*tg*:egfp) wild-type (n = 7) larvae, whereas nearly no EdU-positive cells were detected within the *tg* domain in *isl2a^-/-^* larvae (n = 6). There were also no TUNEL-positive cells (red) in the *tg* domain at 127 hpf. Scale bar = 500 µm. **(D)** Thyroxine (T4) and 3,5,3′-triiodothyronine (T3) levels in the zebrafish body at 44 dpf. Error bars represent ± SD (n = 4). Asterisks indicate significant differences between *isl2a^+/+^* and *isl2a^-/-^* groups (*P < 0.05, **P < 0.01).

As shown in **Figure 3A**, the expression levels of *tg* were reduced in *isl2a^-/-^* eymbryos compared to control embryos both at 3 dpf and 5 dpf, and the trendline of change was similar to that of *tshba*. Notably, *isl2a* ^-/-^ larvae at 5dpf presented reduced mRNA expression of *tshba*, *cga*, *tg* and two other thyroid markers (*tpo* and *slc5a5*) as shown in **Figure 3B**. As detected by WISH, *isl2a^-/-^* larvae present with thyroid hypoplasia. To determine whether the thyroaplasia was due to low TSH level through the pituitary-thyroid axis, we further investigated the mRNA level of thyroid primordium markers (*nkx2.1* and *pax2a*) at 48 hpf when the activating effect of TSH has not yet been fully established. Mutants presented no significant reduction of both early development marker transcripts compared to controls (data not shown), indicating that *isl2a* knockout does not directly affect the development of thyroid primordium.

To further elucidate the involved mechanisms, we analyzed cell proliferation and apoptotic markers in thyroid cells expressing *tg*. Consistent with previously published data reporting that thyrotropin stimulates the proliferation of thyroid cells *in vivo* (34), there were significantly fewer EdU-positive cell fragments within the *tg* domain in 120 hpf *isl2a* ^-/-^ zebrafish’s thyroids (P < 0.05, Student’s t-test) compared to *isl2a* ^+/+^zebrafish, and no statistically relevant difference was observed in TUNEL-positive cell fragments in 127 hpf larvae (**Figures 3C, D**). Overall, these findings suggest that loss of *isl2a* expression impairs zebrafish pituitary development and that thyroaplasia may be induced by low TSH levels through reducing proliferation.

Because thyrotropes defects in *isl2a* mutants during early pituitary development should influence HPT axis function in adults, we used ELISA to compare the whole-body contents of thyroid hormones (THs) in *isl2a* ^-/-^ and *isl2a* ^+/+^ adults. Whole body contents of T4 and T3 in *isl2a* ^-/-^ zebrafish at 44 dpf were significantly lower than those in *isl2a* ^+/+^ zebrafish (**Figure 3D**), indicating that *isl2a* knockout affects thyroid function.

### Developmental phenotype of *isl2a* mutants

**Figure 4:**
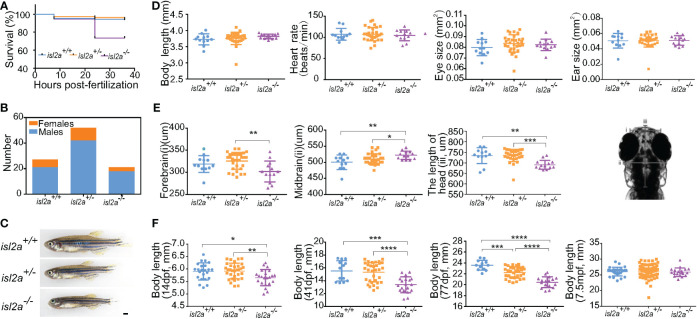
Growth retardation in *isl2a* knockout zebrafish. **(A)** The Kaplan-Meier survival curve of *isl2a^+/+^* (n = 159), *isl2a^+/−^* (n = 173), and *isl2a^−/−^* (n = 253) larvae shows differences. **(B)** Male/female ratios in *isl2a^+/+^* (male/female = 20/6), *isl2a^+/−^* (male/female = 42/10), and *isl2a^−/−^* (male/female = 18/3). **(C)** The *isl2a* variant caused significant growth defects in 41 dpf zebrafish adults. Scale bar = 1 mm. **(D)** Comparison of developmental indicators, including body length, heart rate, eye size, and ear size, among *isl2a^-/-^*, *isl2a^+/+^* and *isl2a^+/-^* siblings at 5 dpf. **(E)** Comparison of three diameter lines of skull among different groups at 5 dpf. **(F)** Dynamic tracking of the body length of *isl2a^-/-^* individuals and their *isl2a^+/+^* and *isl2a^+/-^* siblings at 14 dpf, 41 dpf, 77 dpf, and 7.5 mpf. *P<0.05, **P<0.01, ***P<0.001, ****P<0.0001.

The surviving F3 *isl2a* ^-/-^ embryos were brought to adulthood. In addition to development and growth, we observed a low survival ratio in *isl2a* ^-/-^ individuals. Compared to wild-type fish (86.8% at 7.5 hpf and 84.9% at 24 hpf, n = 159), the survival ratio of *isl2a^-/-^* embryos was 86.9% at 7.5 hpf and 55.0% at 24 hpf (n = 253) (**Figure 4A**). Absence of *isl2a* led to premature death between 0 and 36 hpf, suggesting an essential role for *isl2a* in survival. The female to male ratios were 28.6%, 23.8%, and 16.7% in *isl2a^+/+^*, *isl2a^+/-^*, and *isl2a^-^* ^/-^ adults (**Figure 4B**).

Several development indicators, including body length, heart rate, eye size, and ear size, showed no significant difference between *isl2a* ^-/-^ zebrafish and their *isl2a* ^+/+^ and *isl2a* ^+/-^ siblings at 5 dpf (**Figure 4D**), whereas three diameter lines of the skull showed statistically differences between different groups at 5 dpf (**Figure 4E**). A severe short body length was observed in *isl2a* ^-/-^ larvae from 14 dpf onwards, which progressed during the time course. Measurements taken from different body lengths of *isl2a* ^-/-^ individuals identified significant reductions at 14 dpf, and the reductions were more apparent at 41 dpf when the body size was smaller compared to *isl2a* ^+/+^ and *isl2a* ^+/-^ siblings, with differences visible to the naked eye (**Figures 4C, F**). At 77 dpf, the differences in body length between groups were further enhanced, but the differences disappeared at 7.5 mpf (**Figure 4F**).

In order to determine the effect of low expression levels of *cga* or low thyroid hormones on gonad development between wild-type and *isl2a* mutants, gonads were dissected to extract gonadotropin and steroid hormones, and to conduct histological analyses. Surprisingly, the FSH levels in *isl2a^-/-^* zebrafish gonads were significantly higher than their *isl2a^+/+^* and *isl2a^+/-^* siblings at 42 dpf (**Figure S2A**). However, no significant difference in the LH levels between the three groups (**Figure S2B**). Histological sections of 42dpf *isl2a^-/-^* and *isl2a^+/+^* zebrafish both showed ovary or testis (**Figure S2C**). Moreover, the histological structures of the gonadal tissues were more immature in the *isl2a^-/-^* zebrafish, when compared to that in wild-type zebrafish. Surprisingly, no significant difference was obtained from the levels of testosterone and estradiol between *isl2a^-/-^* and *isl2a^+/+^* zebrafish at around 4mpf (**Figures S3A**, **S3B**). As for secondary sex characteristics of the *isl2a^-/-^* zebrafish, we observed a decrease in vent size in *isl2a^-/-^* female zebrafish at 77dpf compared to that in wild-type female zebrafish (**Figures S3C, S3D**).

## Discussion

In the present study, the expression patterns and functional roles of *ISL2* orthologues (*isl2a* and *isl2b*) in zebrafish were characterized. Using the CRISPR-Cas9 knockout strategy, we discovered that the homozygous variant of *isl2a* caused pituitary and thyroid developmental defects during embryogenesis with reduced expression levels of *tshba*, *cga*, and *tg*. Interestingly, no significant differences in *isl2b* mutants suggested that the low rate of overt phenotypes observed in the zebrafish may be explained by genetic compensation for the effects of loss-of-function variants (35, 36). Moreover, the significantly fewer thyrotrope cells in the pituitary compared to controls as well as the low T3 and T4 levels in *isl2a* homozygous zebrafish were also consistent with central hypothyroidism.

The phenotype of *isl2a* null zebrafish indicates that *ISL2* is involved in thyrotrope development similar to *ISL1*, which is enrichment in thyrotrope lineages in human fetal pituitaries (7) as well as involved in thyrotrope development (12). We hypothesized that *ISL2* and *ISL1* may exert some redundancy in differentiating the pituitary. As we all know, *Lhx3* and *Lhx4 as well as Isl1* and *Isl2* are two such paralogous pairs (37). *Lhx3* and *Isl1* interact inside a well-characterized transcriptional complex that regulates motor neuron development. Indeed, the lack of thyrotropes in *Lhx3* null mice may be due to a delay in *Isl1* expression (12). Interestingly, there is also a *Lhx3*-binding domain in *Isl2* (38). Taken together, we suppose that *ISL2* may form a transcriptional complex with *LHX3* to participate in pituitary development. Besides *ISL1*, several transcription factors, including *POU1F1*, *GATA2*, and *PITX2*, are also required for thyrotrope differentiation (2). Whether *ISL2* acts as a novel regulatory factor or depends on these transcription factors to function in the specification of thyrotropes deserves further study.

Our results agreed with the previous studies on the role of TSH-TSH receptor (TSHR) signaling during thyroid morphogenesis in zebrafish. Knockdown of *tshr* function by morpholino microinjection into embryos causes defects in thyroid later functional differentiation rather than affects early morphogenesis (18). TSH plays an important role in thyroid development by activating the G-protein-coupled TSHR on the surface of the thyroid, but it occurs later than the thyroid primordium development process (39, 40). Taking advantage of this time lag in development, we confirmed that thyroid morphology and function in *isl2a^-/-^* zebrafish are impacted by low TSH levels rather than abnormal thyroid primordium development. Moreover, we found that thyroid hypoplasia is caused by defective thyrotropes through the proliferation pathway. In zebrafish, the *pomca*-expressing cells in the ventral diencephalon are hypothalamic and belong to the arcuate nucleus (41). Due to the distribution of *pomca*-expressing cells in the pituitary and hypothalamus without a significant difference among *isl2a* groups, we presume that hypothalamic development is unaffected. Moreover, the role of the thyrotropin releasing hormone in the regulation of TSH release in zebrafish is less well established. Thus, the possibility of hypothalamic defects or releasing factors secretion contributing to the phenotypes is little.

We discovered that *isl2a^-/-^* zebrafish showed growth retardation during the juvenile stage but reached normal size during adult stage (7.5 mpf) like their control siblings. Significantly, this phenotype is commonly observed in mutant zebrafish with defects in TH signaling, including thyroglobulin (*tg*), dual oxidase (*duox*), and thyroid-stimulating hormone subunit beta a (*tshba*) mutants (44, 42). Compared with the infertility observed in the homozygous *tshba* mutants (44), there were much less severe reproduction defects in our *isl2a^-/-^* fish, just with increased early mortality in embryos. This is due to residual TSH signaling left in the *isl2a* mutants being functional. Nevertheless, decreased TH or TSH signaling cannot fully account for the gender imbalance in our *isl2a* mutants. In previous studies, it was demonstrated that the levels of testosterone were critical to the secondary sex characteristics development (43). Notably, the expression of *cga*, which encodes the α- protein subunits shared by TSH and gonadotrophins, significantly decreased in *isl2a* mutants. The increased proportion of males in *isl2a* mutants is probably attributed to abnormal gonadotrope development and function. In this study, we analyzed gonadal histology and gonadotropin and steroid hormones concentrations. We observed that FSH concentrations were higher in 42 dpf *isl2a^-/-^* zebrafish than their *isl2a^+/+^* and *isl2a^+/-^* siblings, although the gonadal development is delayed. However, the levels of testosterone and estradiol in *isl2a^-/-^* were not decreased compared to that in *isl2a^+/+^* zebrafish at around 4 mpf. Thus, the maturation of gonads in the *tshba* mutants was not totally affected in *isl2a^-/-^* zebrafish, but the morphologic development of gonad was delayed due to growth retardation during the juvenile stage. Moreover, the defective development of secondary sex characteristics was observed in *isl2a^-/-^* zebrafish at 77dpf, caused by impaired TSH signaling in zebrafish as previously reported (32).

The present study has several limitations. First, although downregulation of *isl2a* in zebrafish embryos resulted in HPT axis developmental abnormalities, the mechanisms were not fully elucidated in this study. Further experiments are needed to better understand the molecular events linking this specific regulation of *ISL2* in the HPT axis. Second, the present study did not further explore the impact of *isl2a* on gonadotropes due to the limitation of few homozygotes. Further validations in older adult zebrafish are necessary. Due to the limited data in this study, it is crucial to evaluate the results carefully, especially because the phenotype was subtle. Third, there are inevitable species differences between knockout zebrafish models and humans. Homozygous or compound heterozygous *ISL2* mutations in central hypothyroidism patients have not been detected thus far. Finally, the possibility of the off-target effect in CRISPR-Cas9 applications has not been completely eliminated in our study, albeit we chose a high-scoring guide RNA for our intended target with the silico tool. Despite these limitations, we established the utility of this technique as a tool for human cell biology in pituitary development and disease.

In conclusion, we propose that *ISL2* is important for embryonic pituitary development. In particular, our findings provide insight into the specific regulation of *ISL2* during the terminal differentiation of the pituitary from the lineage cells to thyrotropes. Moreover, *isl2a^-/-^* zebrafish could be used as an alternative *in vivo* model for studying the underlying mechanisms of hypothyroidism diseases and high-throughput screening of potential drugs for treating central hypothyroidism.

## Data availability statement

The original contributions presented in the study are included in the article/**Supplementary Material**. Further inquiries can be directed to the corresponding authors.

## Ethics statement

The animal study was reviewed and approved by the Institutional Animal Care and Use Committee of Shanghai Jiao Tong University School of Medicine.

## Author contributions

C-YY designed and performed the *in vivo* experiments and wrote the draft of the article. F-YW undertook some of the experiments. FS, YF, R-JZ and C-RZ established knockout zebrafish. C-XZ, ZW and LY assisted in the maintenance of zebrafish. R-MY, MD, Q-YZ and X-PYconceived the study and provided experimental guidance. S-XZ and H-DS critically reviewed the manuscript. All authors contributed to the article and approved the submitted version.

## Supplementary material

Supplementary Figure 1 Expression patterns of *isl2b* in zebrafish and no hypothalamus-pituitary-thyroid axis phenotype in mutants. **(A)** Spatiotemporal expression patterns of *isl2b* by WISH at 24 and 48 hpf. At 24 hpf, small black arrows indicate the position of *in situ* hybridization high signals in the midbrain, hindbrain and pharyngeal arch. At 48 hpf, small black arrows indicate the position of high signals in the hindbrain, one endoderm primordium, and branchial arches. Besides, cells in retinal (r) also showed high signals. Scale bars = 200 µm/100 µm. **(B)** Compared to their wild-type and heterozygous siblings, the expression levels of *tshba* and *cga* were unchanged in the *isl2b* mutants. Scale bar = 50 µm. **(C)** Relative levels of *isl2a* transcripts in each *isl2b* genotype group according to qRT-PCR analysis. Error bars represent ± SD (n = 5). **(D)** Expression levels of *tshba* and *cga* in *isl2a* and *isl2b* knockout larvae. Scale bar = 50 µm.

Supplementary Figure 2 Gonadotropic hormone concentrations and histological analysis of gonads in *isl2a* knockout zebrafish. **(A, B)** Follicle-stimulating hormone (FSH) and Luteinizing hormone (LH) concentrations in the zebrafish gonads at 42 dpf. Error bars represent ± SD (n = 5, n= 6, n= 4 in *isl2a^+/+^*, *isl2a^+/-^*, and *isl2a^-/-^*, respectively). Asterisks indicate significant differences between groups (** *P* < 0.01). **(C)** Representative histological sections of gonads from *isl2a^-/-^* and *isl2a^+/+^* zebrafish at 42 dpf by hematoxylin-eosin staining. The gonad can be identified as an ovary by the presence of cortical alveolar oocytes (indicating black arrows) and perinucleolar oocytes (indicating black asterisks). The testis was identified by the presence of lumina filled with sperm, by the clustered organisation (spermatocysts), and by the clusters of spermatogonia. Scale bar = 25 µm.

Supplementary Figure 3 The levels of gonadal hormones (testosterone and estradiol) and changes in secondary sex characteristics in *isl2a^-/-^* zebrafish. **(A)** Quantitative analysis of whole-gonad testosterone contents in *isl2a^-/-^* zebrafish (6 males and 5 females) their wild-type siblings (6 males and 6 females) at 4 mpf by HPLC-MS in positive ion mode. Error bars represent ± SEM. **(B)** Semiquantitative analysis of whole-gonad estradiol contents in *isl2a^-/-^* zebrafish (6 males and 5 females) and their wild-type siblings (6 males and 6 females) at 4 mpf by HPLC-MS in negative ion mode. Error bars represent ± SEM. **(C, D)** Representative female vent in *isl2a^-/-^* and *isl2a^+/^* zebrafish at 77 dpf. Red asterisks indicate the vent anterior to the anal fin. Scale bar = 100 µm. * *P* < 0.05.
