# Supplementary material for: The isl2a transcription factor regulates pituitary development in zebrafish
Source: Front Endocrinol (Lausanne). 2023 Feb 7;14:920548. doi: 10.3389/fendo.2023.920548 (PMC9941339; doi:10.3389/fendo.2023.920548)
Supplement: Supplementary file 1 [file Table_1.docx]

Supplementary Tables

Table S1. Specific primers for genotyping lysed DNA samples.

| Primers | Sequences（5’-3’） |
| --- | --- |
| *isl2a*-geno-F | ACGCCTATTTCTTGCCCCTAT |
| *isl2a*-geno-R | AAACCAGCTAATCTCACGCTG |
| *isl2a*-wt-F | TTGCAGATTATTCGGGAT |
| *isl2a*-wt-R | AAGCGCGTTCCATTAGC |
| *isl2a*-in13-F | ATAAAATAGAGTACGCCTATTTCTTGCC |
| *isl2a*-in13-R | CGTTCCATTAGTCCGTTAGTCCAG |
| *Isl2b* geno-F | TGTGTCCAGAGAAGTCCGG |
| *Isl2b* geno-R | AGAATTGTTGTGGGATAAAATGG |
