# Supplementary material for: The isl2a transcription factor regulates pituitary development in zebrafish
Source: Front Endocrinol (Lausanne). 2023 Feb 7;14:920548. doi: 10.3389/fendo.2023.920548 (PMC9941339; doi:10.3389/fendo.2023.920548)
Supplement: Supplementary file 2 [file Table_2.docx]

Supplementary Tables

Table S2. Primers for WISH probes synthesis.

| Primers | Sequences（5’-3’） |
| --- | --- |
| *isl2a*-ISH-F | GGCCCTTACACATCCCAGAAC |
| *isl2a*-ISH-R | TTACCCAGCGAACCCGACT |
| *isl2b*-ISH-F | GTCCGGGACGAGGAACTGTT |
| *isl2b*-ISH-R | TGGTTGGTAAGCCTGCACATC |
| *tshba-*ISH-F | TTAATGAAGGTTGCCGTGCC |
| *tshba-*ISH-R | CACAGGTTTGGAGCATCTCATC |
| *prl*-ISH-F | TCTCAGCACCTCACTCACCAAT |
| *prl*-ISH-R | TGACAAGTCGAGACGTTTTATCCT |
| *gh*-ISH-F | CTGTTGCAGTTGGTGGTGGTTAGTT |
| *gh*-ISH-R | TCAGGTAGAAATCCTCAAAAGGCAAC |
| *pomca*-ISH-F | GAGCTCAGTGTTGGGAAAACG |
| *pomca*-ISH-R | TTCTTGCGGATAGTCGACCTC |
| *gsu-α/cga*-ISH-F | CGCTGAAGCAAGCATTTTCTT |
| *gsu-α/cga*-ISH-R | AATTGGAAATGGCCACAGTTG |
| *lhx3*-ISH-F | CAGCTCCAGTCAAGATATCCCG |
| *lhx3*-ISH-R | TCGAATCCGCCTCTCTCTGT |
| *lhx4*-ISH-F | ACAGCCAAGCAGAACGATGAT |
| *lhx4*-ISH-R | TCGCCGACACTTCCGTACA |
| *six1b*-ISH-F | TGGCTTAAGGCCCATTACATTG |
| *six1b*-ISH-R | GCGAGAATTCGTCCTCCGA |
| *pit1*-ISH-F | TCTGGCTCCACGTCTCTACAAGT |
| *pit1*-ISH-R | CTCATTAAAAAGAGCGCCGG |
| *pitx3*-ISH-F | CTGGCGGTAGAGGTGATGGAT |
| *pitx3*-ISH-R | TTCTTGCACAGCTCGGCTT |
| *shha*-ISH-F | AGGCTGGATTTGACTGGGTCT |
| *shha*-ISH-R | CATCACCTTTTGTCCGGCTC |
