# Supplementary material for: The isl2a transcription factor regulates pituitary development in zebrafish
Source: Front Endocrinol (Lausanne). 2023 Feb 7;14:920548. doi: 10.3389/fendo.2023.920548 (PMC9941339; doi:10.3389/fendo.2023.920548)
Supplement: Supplementary file 3 [file Table_3.docx]

Supplementary Tables

Table S3. Primers sequences for real-time PCR.

| Primers | Sequences（5’-3’） |
| --- | --- |
| zebrafish-*β-actin*-F | ATGGATGAGGAAATCGCTGCC |
| zebrafish-*β-actin*-R | CTCCCTGATGTCTGGGTCGTC |
| zebrafish- *ef1α* -F | AAGACAACCCCAAGGCTCTCA |
| zebrafish- *ef1α*-R | CCTTTGGAACGGTGTGATTGA |
| zebrafish-*isl2a*-F | GAGTTCTCATTGCGGGACGA |
| zebrafish-*isl2a*-R | CTGGCACAGGTTCTGGGATG |
| zebrafish-*isl2b*-F | CAGACTGAAGTTGCGGGACA |
| zebrafish-*isl2b*-R | GGACTTCTTTTTGGAATGATCCCC |
| zebrafish-*tshba*-F | CAATTACTGTGTGGCTGTCAAC |
| zebrafish-*tshba*-R | CCGGTACTCAACTTCCTGATAA |
| zebrafish-*cga*-F | TCCGTCTATCAGTGCGT |
| zebrafish-*cga*-R | GGATATTCGTGGCAACCATTT |
| zebrafish-*tg*-F | CTTACCTGAAAACGCTGCTTAT |
| zebrafish-*tg*-R | GGGTGAATGTTTTCTCCTTGTA |
| zebrafish-*slc5a5*-F | ACAAAAGCCCTGCGATGC |
| zebrafish-*slc5a5*-R | TTGTTTATCAGCAGCAATCGGA |
| zebrafish-*tpo*-F | AGCCCCTGGAGACTCGTCC |
| zebrafish-*tpo*-R | CTCGTCCTCTCTGTAGGTTCAGC |
