# Supplementary figures and images for: The isl2a transcription factor regulates pituitary development in zebrafish
Source: Front Endocrinol (Lausanne). 2023 Feb 7;14:920548. doi: 10.3389/fendo.2023.920548 (PMC9941339; doi:10.3389/fendo.2023.920548)

**A**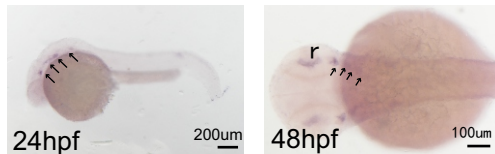**B**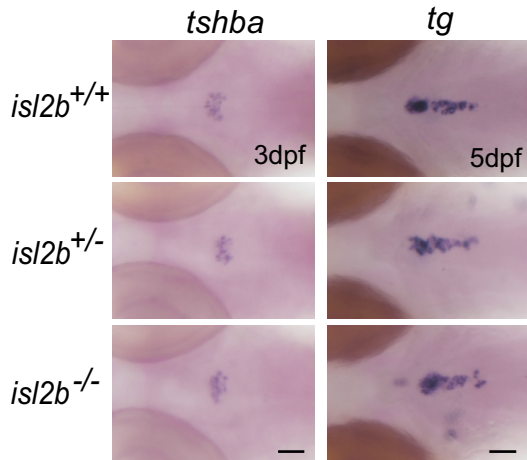**C**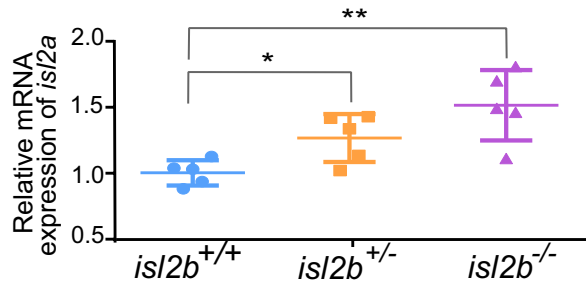**D**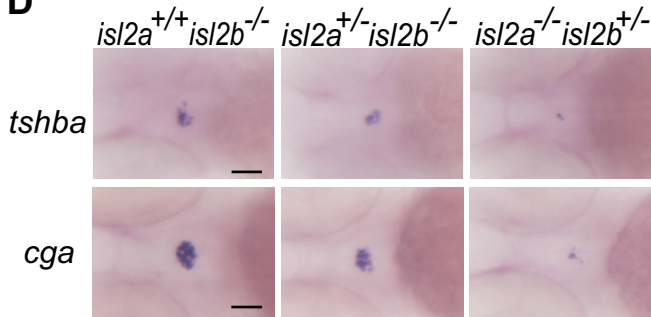

Supplement: Supplementary Figure 1 — Expression patterns of isl2b in zebrafish and no hypothalamus-pituitary-thyroid axis phenotype in mutants. (A) Spatiotemporal expression patterns of isl2b by WISH at 24 and 48 hpf. At 24 hpf, small black arrows indicate the position of in situ hybridization high signals in the midbrain, hindbrain and pharyngeal arch. At 48 hpf, small black arrows indicate the position of high signals in the hindbrain, one endoderm primordium, and branchial arches. Besides, cells in retinal (r) also showed high signals. Scale bars = 200 µm/100 µm. (B) Compared to their wild-type and heterozygous siblings, the expression levels of tshba and cga were unchanged in the isl2b mutants. Scale bar = 50 µm. (C) Relative levels of isl2a transcripts in each isl2b genotype group according to qRT-PCR analysis. Error bars represent ± SD (n = 5). (D) Expression levels of tshba and cga in isl2a and isl2b knockout larvae. Scale bar = 50 µm. [file Image_1.pdf]

**A**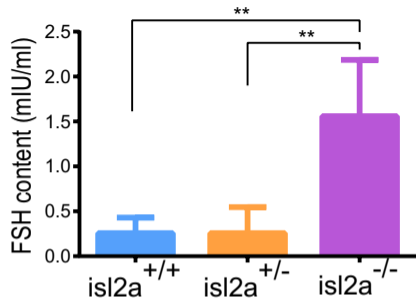**B**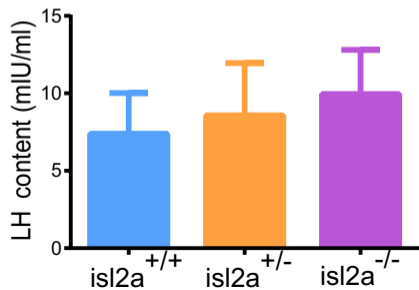**C**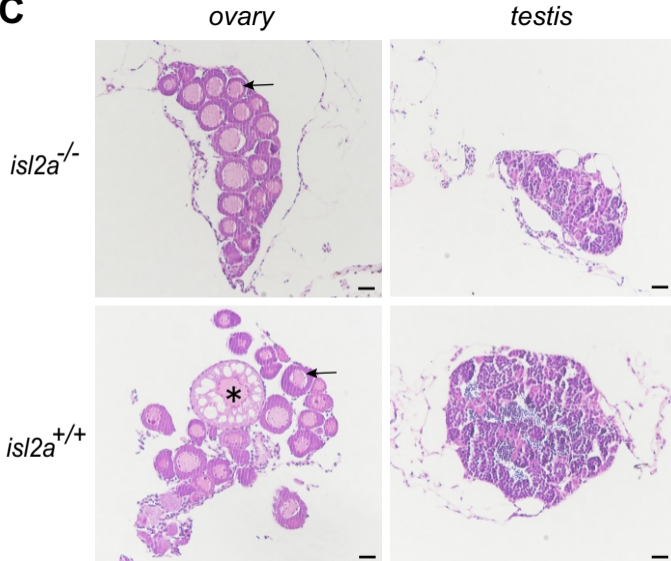

Supplement: Supplementary Figure 2 — Gonadotropic hormone concentrations and histological analysis of gonads in isl2a knockout zebrafish. (A, B) Follicle-stimulating hormone (FSH) and Luteinizing hormone (LH) concentrations in the zebrafish gonads at 42 dpf. Error bars represent ± SD (n = 5, n= 6, n= 4 in isl2a+/+ , isl2a+/- , and isl2a-/- , respectively). Asterisks indicate significant differences between groups (** P < 0.01). (C) Representative histological sections of gonads from isl2a-/- and isl2a+/+ zebrafish at 42 dpf by hematoxylin-eosin staining. The gonad can be identified as an ovary by the presence of cortical alveolar oocytes (indicating black arrows) and perinucleolar oocytes (indicating black asterisks). The testis was identified by the presence of lumina filled with sperm, by the clustered organisation (spermatocysts), and by the clusters of spermatogonia. Scale bar = 25 µm. [file Image_2.pdf]

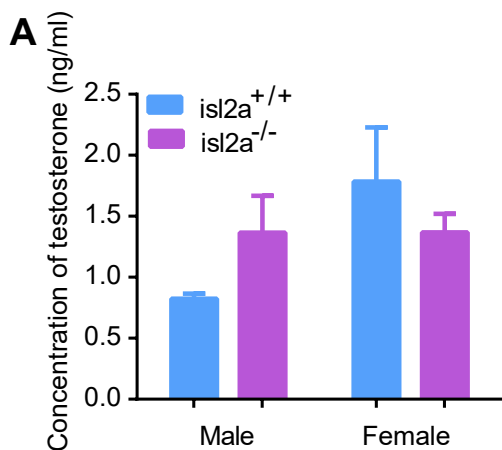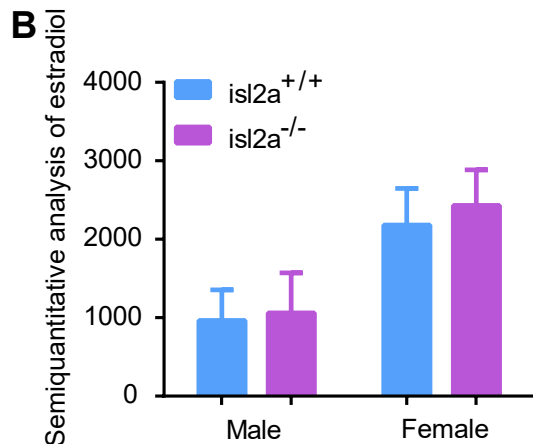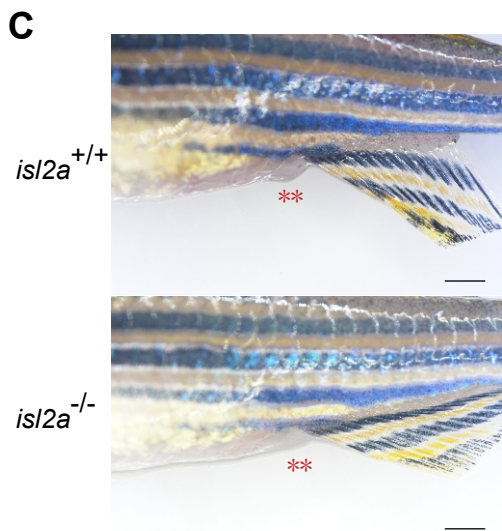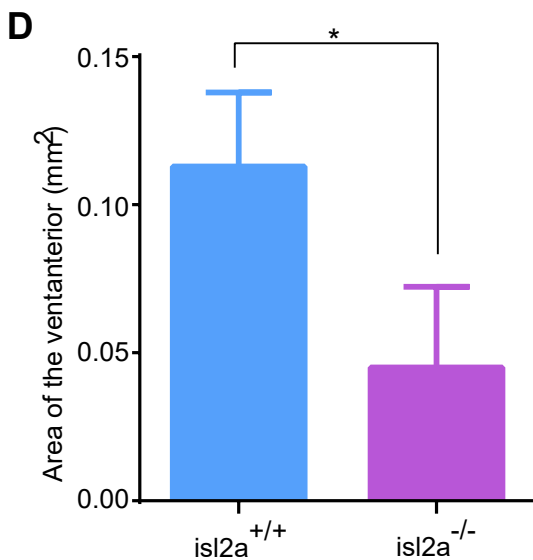

Supplement: Supplementary Figure 3 — The levels of gonadal hormones (testosterone and estradiol) and changes in secondary sex characteristics in isl2a-/- zebrafish. (A) Quantitative analysis of whole-gonad testosterone contents in isl2a-/- zebrafish (6 males and 5 females) their wild-type siblings (6 males and 6 females) at 4 mpf by HPLC-MS in positive ion mode. Error bars represent ± SEM. (B) Semiquantitative analysis of whole-gonad estradiol contents in isl2a-/- zebrafish (6 males and 5 females) and their wild-type siblings (6 males and 6 females) at 4 mpf by HPLC-MS in negative ion mode. Error bars represent ± SEM. (C, D) Representative female vent in isl2a-/- and isl2a+/ zebrafish at 77 dpf. Red asterisks indicate the vent anterior to the anal fin. Scale bar = 100 µm. * P < 0.05. [file Image_3.pdf]
